# Supplementary material for: Perfluoroalkyl Substances (PFASs) in Rivers and Drinking Waters from Qingdao, China
Source: Int J Environ Res Public Health. 2022 May 8;19(9):5722. doi: 10.3390/ijerph19095722 (PMC9104605; doi:10.3390/ijerph19095722)
Supplement: Supplementary file 1 [file ijerph-19-05722-s001.zip › ijerph-1667801-supplementary.pdf]

Supplementary Information-Table S1-S4

Table S1. Information on surface water samples in Qingdao.

Field No.	Latitude	Longitude	Location	Description
W1	36°05′47″	120°22′43″	The Haibo River, Liaoyuan Road, Shibei District	Residential area
W2	36°06′16″	120°20′03″	The Haibo River, Hai'an Road, Shibei District	Residential area
W3	36°06′27″	20°19′42″	The Haibo River, Hang'an Road, Shibei District	Harbor
W4	36°07′30″	120°26′03″	The Zhangcun River, Zhuzhou Road, Laoshan District	Sourounded by furniture workshops
W5	36°08′29″	120°28′47″	The Zhangcun River, Songling Road, Laoshan District	Sub-urban area
W6	36°09′56″	120°30′4″	The Zhangcun River, Jiushui East Road, Laoshan District	Near China Ocean University
W7	36°11′14″	120°30′53″	The Zhangcun River, Laoshan District	Sub-urban area
W8	36°11′56″	120°32′5″	Kuang Chuan Reservoir, Laoshan District	Drinking water source, upper end of the Zhangcun River
W9	36°11′3″	120°28′55″	The Licun River, Licun River Park, Licang District	Near a public park
W10	36°11′1″	120°28′10″	The Licun River, Licang District	Rural area
W11	36°9′49″	120°28′4″	The Licun River, Licang District	Rural area
W12	36°9′39″	120°26′55″	The Licun River, Licang District	Rural area. Sourounded by villages
W13	36°15′24″	120°28′20″	Laoshan Reservoir, Chengyang District	Drinking water source site
W14	36°14′57″	120°23′16″	The Baisha river, Chengyang District	Rural area
W15	36°15′59″	120°24′57″	The Baisha river, Chengyang District	Rural area
W16	36°15′12″	120°20′8″	The Baisha river, Chengyang District	Rural area. Surrounded by villages
W17	36°21′13″	120°24′59″	The Moshui River, Chengyang District	Rural area.
W18	36°18′38″	120°20′59″	The Moshui River, Chengyang District	Rural area with plastic & printing factories
W19	36°17′30″	120°19′27″	The Moshui River, Chengyang District	Rural area.
W20	36°16′25″	120°19′19″	The Moshui River, Chengyang District	Rural area influenced by the seawater flowing backward
W21	36°15′48″	120°17′41″	The Hongjiang River Estuary, Chengyang District	Rural area influenced by the seawater flowing backward
W22	36°17′21″	120°17′5″	The Hongjiang River, Chengyang District	Rural area.

W23	36°09′0″	120°24′46″	The Licun River,, Licang District	Residential area with nearly stagnant water flow
W24	36°09′3″	120°22′29″	The Licun River, Shibei District	The entrance of the Jiaozhou Bay Cross-sea Bridge
W25	36°09′12″	120°22′47″	The Licun River, Shibei District	Under the land part of the Jiaozhou Bay Cross-sea Bridge
W26	36°08′25″	120°23′56″	The Zhangcun River, Shibei District	Near a WWTP
W27	36°08′47″	120°23′51″	The Licun River, Shibei District, joint with the Zhangcun River	Near a WWTP

Table S2: Information on drinking water and groundwater samples from Qingdao

Field No.	Sample type	Sampling sites	Producers and brand
TW1	Tapwater	90 Dunhua Road, Carrefour, Shibei District	Qingdao Water Group Co. Ltd.
TW2	Tapwater	Longkou Village, Jiushui East Road, Laoshan District	Qingdao Water Group Co. Ltd.
TW3	Tapwater	Licun River Park, Licang District	Qingdao Water Group Co. Ltd.
TW4	Tapwater	20 Xiata Road, Chengyang District	Qingdao Water Group Co. Ltd.
TW5	Tapwater	Campus of Qingdao Agriculture University, Chengyang District	Qingdao Water Group Co. Ltd.
TW6	Tapwater	308 Ningxia Road, Qingdao University, Shinan District	Qingdao Water Group Co. Ltd.
TW7	Tapwater	Beach Park, Chongming Island Road, Huangdao District	Qingdao Water Group Co. Ltd.
MW1	Mineral water	90 Dunhua Road, Carrefour, Shibei District	Red bottle, Laoshan Mineral Water Co. Ltd.
MW2	Mineral water	90 Dunhua Road, Carrefour, Shibei District	Blue bottle, Laoshan Mineral Water Co. Ltd.
MW3	Mineral water	90 Dunhua Road, Carrefour, Shibei District	Lanke Mineral Water Co. Ltd.
BW1	Barreled pure water	Qing-Yin Highway Bridge, Shenzhen Road, Laoshan District	Produced in local residential quarters
BW2	Barreled pure water	369 Hexing Road, Laoshan Distrct	Produced in local residential quarters
BW3	Barreled pure water	Qingdao Agriculture University, Chengyang District	Produced by Qingdao Ganlu Spring Co. Ltd.
BW4	Barreled pure water	59 Hexing Road, Wangjia Plaza, Shibei District	Produced in restaurants
BW5	Barreled pure water	Huangdingshan Road, Huangdao District	Produced by Laoshan Branch, Qingdao Zhichun Baverage
BW6	Barreled pure water	308 Ningxia Road, Qingdao University, Shinan District	Qingdao Beer & Beverage Co. Ltd.

GW1	Groundwater	Qingdao Jinrui Wood Co. Ltd., Shenzhen Road, Laoshan District	Depth：10 m.　Not use for drinking
GW2	Groundwater	396 Songling Road, Laoshan District	Depth：6 m.　Not used for drinking
GW3	Groundwater	995 Xiazhuang Village, Chengyang District	Depth：9 m.　Used for drinking

Table S3 The precursor and product ions in mass spectrometry, the limits of quantification (LOQs), recoveries, and the repeatability of matrix spike recoveries for individual PFASs in water samples

Compounds	Precursor ions	Product ions	Blank (ng/L)		LOQ (ng/L)	Matrix Recovery (n=11)	
	(m/z)	(m/z)	Travelling	Procedure		Mean (%)	RSD (%)
PFBA	212.8	212.8	<0.05	<0.1	0.1	93.9	4.8
PFPeA	262.8	262.8	<0.05	<0.1	0.2	85.9	3.0
PFHxA	313	313	<0.05	<0.1	0.2	87.0	3.3
PFHpA	363	363	<0.05	<0.1	0.5	97.1	3.6
PFOA	412.8	412.8	<0.05	<0.1	0.1	86.8	2.4
PFNA	462.8	462.8	<0.05	<0.1	0.1	88.3	1.8
PFDA	512.8	512.8	<0.05	<0.1	0.5	90.1	1.4
PFUnDA	562.8	562.8	<0.05	<0.1	0.2	87.4	1.8
PFDoDA	612.8	612.8	<0.05	<0.1	0.5	83.6	2.9
PFTrDA	662.8	662.8	<0.05	<0.1	0.5	81.5	3.2
PFTeDA	712.8	712.8	<0.05	<0.1	0.2	80.5	3.6
PFHxDA	813	813	<0.05	<0.1	0.2	79.9	5.2
PFOcDA	912.8	912.8	<0.05	<0.1	0.2	72.4	3.8
PFBS	298.8	298.8	<0.1	<0.2	0.5	93.4	2.2
PFHxS	398.8	398.8	<0.1	<0.2	0.5	93.7	0.9
PFOS	499	499	<0.1	<0.2	0.5	97.8	4.4
PFDS	598.8	598.8	<0.1	<0.2	0.5	79.4	3.0
PFOSA	498	77.8	<0.05	<0.1	0.05	80.7	4.3

RSD: relative standard deviation

Table S4 Concentrations of PFASs in the water samples (ng/L)

Water type	Field No.	PFBA	PFPeA	PFHxA	PFOA	PFHpA	PFNA	PFDA	PFUnD	PFOS	PFHxS	PFBS	PFTrD	PFDoD	PFTeD	PFHxD	PFOcD	PFDS	PFOSA	ΣPFASs
River water																				
The Haibo River	W1	17.60	3.91	4.04	19.44	2.12	0.39	<0.5	<0.2	2.24	2.28	50.40	<0.5	<0.5	<0.2	<0.2	<0.2	<0.5	<0.1	**102.6**
	W2	21.00	4.60	3.79	38.00	2.54	0.42	<0.5	<0.2	5.00	9.40	36.60	<0.5	<0.5	<0.2	<0.2	<0.2	<0.5	3.12	**124.7**
	W3	8.68	3.22	2.52	15.36	1.36	0.27	<0.5	<0.2	1.06	2.36	13.88	<0.5	<0.5	<0.2	<0.2	<0.2	<0.5	0.28	**49.0**
The Zhangcun	W7	12.64	3.90	2.91	11.04	1.60	<0.1	<0.5	<0.2	0.00	33.84	11.40	<0.5	<0.5	<0.2	<0.2	<0.2	<0.5	<0.1	**77.4**
	W6	13.68	2.42	2.16	12.64	2.07	0.34	<0.5	<0.2	0.54	1.34	**256.8**	<0.5	<0.5	<0.2	<0.2	<0.2	<0.5	<0.1	**292.2**
	W5	12.12	2.77	2.39	14.56	2.81	0.54	<0.5	<0.2	0.78	1.23	51.20	<0.5	<0.5	<0.2	<0.2	<0.2	<0.5	<0.1	**88.6**
	W4	19.92	3.39	2.84	23.40	2.44	0.52	<0.5	<0.2	1.24	1.85	32.40	<0.5	<0.5	<0.2	<0.2	<0.2	<0.5	<0.1	**88.3**
	W26	22.52	2.64	5.16	7.04	4.88	<0.1	<0.5	<0.2	0.51	1.00	7.88	<0.5	<0.5	<0.2	<0.2	<0.2	<0.5	<0.1	**51.8**
The Licun River	W9	10.00	1.40	1.13	8.88	1.52	0.20	<0.5	<0.2	0.06	0.40	4.64	<0.5	<0.5	<0.2	<0.2	<0.2	<0.5	<0.1	**28.3**
	W10	17.52	3.33	2.79	24.00	4.04	0.56	<0.5	<0.2	0.30	0.99	8.24	<0.5	<0.5	<0.2	<0.2	<0.2	<0.5	<0.1	**62.1**
	W11	17.64	3.43	3.30	21.60	4.04	0.70	<0.5	<0.2	0.66	1.59	15.40	<0.5	<0.5	<0.2	<0.2	<0.2	<0.5	<0.1	**68.7**
	W12	18.56	4.20	3.32	27.20	3.65	1.08	<0.5	<0.2	1.39	2.21	20.80	<0.5	<0.5	<0.2	<0.2	<0.2	<0.5	<0.1	**82.9**
	W23	19.76	7.36	6.96	36.40	11.72	3.16	2.46	0.60	3.65	3.67	19.60	<0.5	<0.5	<0.2	<0.2	<0.2	<0.5	0.92	**116.3**
	W27	**41.60**	4.44	8.84	15.92	5.28	0.32	<0.5	0.23	1.36	0.86	11.72	<0.5	<0.5	<0.2	<0.2	<0.2	<0.5	<0.1	**90.9**
	W25	17.88	3.93	3.58	22.60	3.26	0.89	0.58	<0.2	1.10	5.80	40.00	<0.5	<0.5	<0.2	<0.2	<0.2	<0.5	<0.1	**99.6**
	W24	13.36	4.68	3.77	<0.1	<0.5	<0.1	<0.5	<0.2	0.76	0.04	17.08	<0.5	<0.5	<0.2	<0.2	<0.2	<0.5	<0.1	**39.8**
The Baisha River	W15	15.68	4.76	4.00	15.00	3.66	0.92	0.58	0.30	2.06	0.92	17.40	<0.5	<0.5	<0.2	<0.2	<0.2	<0.5	<0.1	**65.3**
	W14	19.32	6.24	4.40	72.40	4.96	1.30	0.52	<0.2	2.03	0.61	14.36	<0.5	<0.5	<0.2	<0.2	<0.2	<0.5	<0.1	**126.1**
	W16	10.28	7.60	5.56	29.20	4.88	1.15	0.77	0.25	9.92	1.37	22.80	<0.5	<0.5	<0.2	<0.2	<0.2	<0.5	0.51	**94.3**
The Moshui River	W17	22.80	22.80	30.20	57.80	12.72	5.76	4.84	0.95	15.48	3.35	25.40	<0.5	<0.5	<0.2	<0.2	<0.2	<0.5	<0.1	**202.1**

	W18	26.20	24.84	27.80	68.40	13.68	5.52	4.20	0.71	15.28	3.44	27.96	<0.5	<0.5	<0.2	<0.2	<0.2	<0.5	<0.1	**218.0**
	W19	30.32	10.28	9.72	55.20	6.48	1.56	0.92	0.29	26.96	5.56	32.72	<0.5	<0.5	<0.2	<0.2	<0.2	<0.5	0.47	**180.5**
	W20	8.84	7.56	8.20	21.60	3.68	1.31	0.69	0.25	3.78	0.82	7.88	<0.5	<0.5	<0.2	<0.2	<0.2	<0.5	0.29	**64.9**
The Hongjiang	W22	27.24	32.32	41.20	32.48	19.64	3.94	1.93	0.30	41.60	5.20	15.24	<0.5	<0.5	<0.2	<0.2	<0.2	<0.5	44.0	**265.1**
	W21	24.96	18.20	25.00	33.24	15.44	2.80	1.06	0.24	25.96	5.00	16.08	<0.5	<0.5	<0.2	<0.2	<0.2	<0.5	13.8	**181.8**
Groundwater	GW1	5.26	0.35	0.19	3.32	0.33	0.32	<0.5	<0.2	4.84	0.48	4.44	<0.5	<0.5	<0.2	<0.2	<0.2	<0.5	<0.1	**19.5**
	GW2	1.40	0.20	0.20	0.85	0.33	<0.1	<0.5	<0.2	0.43	0.00	1.52	<0.5	<0.5	<0.2	<0.2	<0.2	<0.5	<0.1	**4.9**
	GW3	0.20	<0.2	<0.2	<0.1	<0.5	<0.1	<0.5	<0.2	0.00	0.00	0.09	<0.5	<0.5	<0.2	<0.2	<0.2	<0.5	<0.1	**0.4**
Reservoir	W8	16.72	2.39	2.08	12.96	2.77	0.74	<0.5	<0.2	0.10	0.00	1.51	<0.5	<0.5	<0.2	<0.2	<0.2	<0.5	<0.1	**39.4**
	W13	8.44	2.43	1.74	7.48	1.58	0.37	<0.5	<0.2	0.47	1.20	4.72	<0.5	<0.5	<0.2	<0.2	<0.2	<0.5	<0.1	**28.6**
Tapwater	TW1	5.32	1.53	1.25	6.24	1.03	0.30	<0.5	<0.2	1.09	3.04	1.08	<0.5	<0.5	<0.2	<0.2	<0.2	<0.5	<0.1	**20.9**
	TW2	5.16	1.68	1.39	7.66	1.42	0.40	<0.5	<0.2	1.60	4.50	1.62	<0.5	<0.5	<0.2	<0.2	<0.2	<0.5	<0.1	**25.4**
	TW3	5.80	2.04	1.52	9.64	1.65	0.52	<0.5	<0.2	2.32	4.74	1.63	<0.5	<0.5	<0.2	<0.2	<0.2	<0.5	<0.1	**29.9**
	TW4	5.06	1.80	1.20	6.04	1.18	0.30	<0.5	<0.2	1.91	1.87	2.34	<0.5	<0.5	<0.2	<0.2	<0.2	<0.5	<0.1	**21.7**
	TW5	5.02	1.57	1.23	8.46	1.35	0.35	<0.5	<0.2	1.71	4.22	1.58	<0.5	<0.5	<0.2	<0.2	<0.2	<0.5	<0.1	**25.5**
	TW6	4.62	1.44	1.18	6.80	1.26	0.31	<0.5	<0.2	1.25	2.26	1.41	<0.5	<0.5	<0.2	<0.2	<0.2	<0.5	<0.1	**20.5**
	TW7	5.10	1.75	1.36	9.62	1.32	0.45	<0.5	<0.2	2.16	4.14	1.92	<0.5	<0.5	<0.2	<0.2	<0.2	<0.5	<0.1	**27.8**
Barreled pure water	BW1	0.27	<0.2	<0.2	<0.1	<0.5	<0.1	<0.5	<0.2	0.00	0.00	0.00	<0.5	<0.5	<0.2	<0.2	<0.2	<0.5	<0.1	**0.3**
	BW2	0.18	<0.2	<0.2	<0.1	<0.5	<0.1	<0.5	<0.2	0.00	0.00	0.00	<0.5	<0.5	<0.2	<0.2	<0.2	<0.5	<0.1	**0.2**
	BW3	0.40	<0.2	<0.2	<0.1	<0.5	<0.1	<0.5	<0.2	0.00	0.00	0.00	<0.5	<0.5	<0.2	<0.2	<0.2	<0.5	<0.1	**0.8**
	BW4	5.14	1.65	1.36	9.52	1.64	0.48	<0.5	<0.2	1.95	4.76	1.89	<0.5	<0.5	<0.2	<0.2	<0.2	<0.5	<0.1	**28.4**
	BW5	6.32	1.91	1.17	6.10	1.04	0.25	<0.5	<0.2	1.11	2.94	1.39	<0.5	<0.5	<0.2	<0.2	<0.2	<0.5	<0.1	**22.2**

	BW6	0.92	0.00	0.00	<0.1	<0.5	<0.1	<0.5	<0.2	0.00	0.00	0.00	<0.5	<0.5	<0.2	<0.2	<0.2	<0.5	<0.1	**1.1**
Bottled mineral water	MW1	0.22	<0.2	<0.2	<0.1	<0.5	<0.5	<0.5	<0.2	0.00	0.00	0.00	<0.5	<0.5	<0.2	<0.2	<0.2	<0.5	<0.1	**0.3**
	MW2	0.22	<0.2	<0.2	<0.1	<0.5	<0.5	<0.5	<0.2	0.00	0.00	0.00	<0.5	<0.5	<0.2	<0.2	<0.2	<0.5	<0.1	**0.3**
	MW3	4.28	1.78	0.58	<0.1	0.16	<0.5	<0.5	<0.2	0.00	0.00	0.38	<0.5	<0.5	<0.2	<0.2	<0.2	<0.5	<0.1	**7.2**
